# Different extrapolation of moving object locations in perception, smooth pursuit, and saccades

**DOI:** 10.1167/jov.24.3.9

**Published:** 2024-03-28

**Authors:** Matteo Lisi, Patrick Cavanagh

**Affiliations:** 1 Department of Psychology, Royal Holloway, University of London, London, UK; 2 Department of Psychology, Glendon College, Toronto, Ontario, Canada; 3 Department Psychological and Brain Sciences, Dartmouth College, Hanover, NH, USA

**Keywords:** motion-2D, position, saccades, smooth pursuit, illusion

## Abstract

The ability to accurately perceive and track moving objects is crucial for many everyday activities. In this study, we use a “double-drift stimulus” to explore the processing of visual motion signals that underlie perception, pursuit, and saccade responses to a moving object. Participants were presented with peripheral moving apertures filled with noise that either drifted orthogonally to the aperture’s direction or had no net motion. Participants were asked to saccade to and track these targets with their gaze as soon as they appeared and then to report their direction. In the trials with internal motion, the target disappeared at saccade onset so that the first 100 ms of the postsaccadic pursuit response was driven uniquely by peripheral information gathered before saccade onset. This provided independent measures of perceptual, pursuit, and saccadic responses to the double-drift stimulus on a trial-by-trial basis. Our analysis revealed systematic differences between saccadic responses, on one hand, and perceptual and pursuit responses, on the other. These differences are unlikely to be caused by differences in the processing of motion signals because both saccades and pursuits seem to rely on shared target position and velocity information. We conclude that our results are instead due to a difference in how the processing mechanisms underlying perception, pursuit, and saccades combine motor signals with target position. These findings advance our understanding of the mechanisms underlying dissociation in visual processing between perception and eye movements.

## Introduction

Visual motion serves multiple functions in visual perception, including object tracking, foreground–background segmentation (Born, Groh, Zhao, & Lukasewycz, 2000), depth perception through motion parallax (Rogers & Graham, 1979), and computation of three-dimensional (3D) structure (Jain & Zaidi, 2011). Beyond perception, visual motion plays a critical role in motor control, such as smooth pursuit eye movements (Lisberger, 2010), online adjustments of hand movements (Gomi, 2008; Whitney, Westwood, & Goodale, 2003), and posture control (Kelly, Loomis, & Beall, 2005; Lee & Lishman, 1975). Estimating the velocity of moving objects is crucial for accurate planning of actions directed toward objects in motion. Even saccadic eye movements, the fastest goal-directed action that human and nonhuman primates can produce, are influenced by velocity information when directed toward a moving object (Etchells, Benton, Ludwig, & Gilchrist, 2010; Gellman & Carl, 1991; Groh, Born, & Newsome, 1997; Quinet & Goffart, 2015).

When tracking moving objects, the brain uses motion signals to correct for the displacement of targets and guide interceptive motor actions. Evidence suggests that similar corrective adjustments are implemented in perception, where moving objects are usually perceived ahead of their physical location in space (De Valois & De Valois, 1991; Ramachandran & Anstis, 1990; Whitney, 2002). However, whether perception and movement are guided by the same visual processing mechanisms is a topic of debate (Cardoso-Leite & Gorea, 2010; Spering & Carrasco, 2015).

In a previous study, we investigated the processes that determine the perceived location of a moving object and those guiding saccadic eye movements toward the same object (Lisi & Cavanagh, 2015). We found that these processes are different. Participants were asked to make eye movements and judge the location of a moving stimulus whose perceived and physical direction of motion were dissociated, producing systematic errors in its perceived location. The stimulus was a moving aperture containing a drifting pattern that moved along a direction 90° apart from the physical direction of displacement of the aperture (a double-drift stimulus). While perceptual judgments revealed the accumulation of a location error along the perceived (as opposed to physical) direction of motion over a long interval (Kwon, Tadin, & Knill, 2015), interceptive eye movements were much less influenced by the perceived motion direction and showed only a small, constant error that did not vary over time. We found that the perceived trajectory could be recovered from the distributions of saccade landing positions only when a delay was introduced between the presentation of the stimulus and the onset of the movement, so that saccadic responses were memory-guided rather than based on current visual input (Massendari, Lisi, Collins, & Cavanagh, 2018). This suggests that the visual information that guides saccades closer to the physical than the perceived location must be short-lived, possibly related to the generally fast decay of neural activity observed in oculomotor structures after the disappearance of static visual targets (Edelman & Goldberg, 2001) (although there are instances of maintained discharge under some conditions; see Mays & Sparks 1980; Sparks, Rohrer, & Zhang 2000). Indeed, we found that the dissociation between saccade landing and perceived location was limited to interceptive saccades. Hand-pointing movements, even when the pointing latency was matched to that of eye movements, revealed a systematic bias toward the perceived trajectory (Lisi & Cavanagh, 2017c).

Saccades are not the only eye movements that differ from perception. Pursuit eye movements, in some cases, reveal motion estimates that deviate from perceptual judgments (Simoncini, Perrinet, Montagnini, Mamassian, & Masson, 2012; Spering, Pomplun, & Carrasco, 2011; Tavassoli & Ringach, 2010). To compare motion estimates used in saccades, pursuit, and perception, we developed a novel experimental paradigm that allowed us to measure pursuit, perceptual, and saccadic responses to a peripheral double-drift stimulus simultaneously. Our results indicate that perceptual direction judgments and pursuit responses have a strong similarity, as they both display large deviations from the physical direction of the target. On the other hand, saccade landing positions show only a small bias, which suggests either extrapolation along a direction closer to the physical direction of the target or extrapolation along the perceived direction but for a shorter interval than the saccadic latency. Saccadic and pursuit eye movements are usually interdependent and cooperate to track moving objects (Erkelens, 2006; Goettker, Braun, & Gegenfurtner, 2019; Goettker, Braun, Schütz, & Gegenfurtner, 2018; Lisi & Cavanagh, 2017a), so it is likely that they both depend on the same motion signals. Thus, our results suggest that the dissociations between saccades and perceptual judgments in the localization of moving objects (Lisi & Cavanagh, 2015) are not due to differences in the motion signals available to the two systems but to differences in how those motion signals are used to estimate future target positions.

## Material and methods

### Participants

Eight subjects participated in Experiment 1 (two males, one the author and six females; age range 24–35). A separate group of four subjects took part in Experiment 2 (two males and two females; age range 32–38); all subjects were compensated 10€ per hour. All had normal or corrected-to-normal vision and gave their informed consent to perform the experiments. The study was conducted in accordance with French regulations and the requirements of the Helsinki convention. All participants had prior experience with psychophysical experiments and (except the author) were naive to the specific purpose of the experiment. The data supporting the findings of this study are available in the Open Science Framework repository at the following link: https://osf.io/57hdm/.

### Setup

Participants sat in a quiet, dark room. We recorded the right-eye gaze position with an SR-Research Eyelink 1000 desktop mount, at a sampling rate of 1 kHz. In Experiment 1, participants had their heads positioned on a chinrest, with adjustable forehead rest, at 180 cm in front of a white projection screen. A PROPixx DLP LED projector placed behind the participants and above their head was used to project the stimuli onto the screen at 120 Hz. The image projected onto the screen was 137.5 cm wide (resolution 1280 × 720), covering ≈42 degrees of visual angle (dva). The same setup was used in Experiment 2, except that the chinrest was now placed at 120 cm from the projection screen (which then covered ≈60 dva), and the resolution of the image was 1,600 × 900 (the stimuli were scaled to cover approximately the same area in retinal space). An Apple computer running MATLAB (MathWorks) with the Psychophysics and Eyelink toolboxes controlled stimulus presentation and response collection in both experiments.

### Stimuli

Stimuli were noise patterns presented within a Gaussian contrast envelope moving at 12 dva/s. The noise pattern within the envelope could either drift in a direction orthogonal to that of the envelope (double-drift stimulus) or vary randomly with no net motion (control stimulus). Double-drift stimuli were generated by taking square snapshots from a two-dimensional (2D) surface of 1/*f* luminance noise. In Experiment 1, each snapshot was shifted 2 pixels from the previous one along a constant direction, resulting in a speed of ≈8 dva/s. Control stimuli were generated by taking square 2D snapshots from a 3D volume of 1/*f* noise, with each snapshot shifted in depth by 1 pixel from the previous one (this value was chosen to match the perceived temporal frequency of the double-drift stimulus on the basis of preliminary tests; the results of the discriminability task confirmed a posteriori that this value made the two stimuli perceptually similar when seen in the periphery). Since the 3D noise volume was anisotropic, the internal pattern in the control stimulus varied but did not contain any prevalent direction, and therefore differently from the double-drift stimulus, it did not induce any systematic distortion of the motion direction. In Experiment 2, double-drift stimuli with different internal speeds were obtained by shifting the noise pattern by 1, 2, or 3 pixels (corresponding to ≈5, ≈10, and ≈15 dva/s, respectively); control stimuli had also three different temporal rates, created by taking subsequent 2D snapshots in depth at half the rate of the double-drift stimulus (obtained by interpolating the noise pattern for distances of a half pixel). Each snapshot was multiplied with a 2D Gaussian with *SD* set to 0.35 dva and constituted one frame (≈8.3 ms) of stimulus presentation. The noise patterns were generated randomly at the beginning of each trial.

### Procedure

#### Experiment 1: Main task

**Figure 1.**
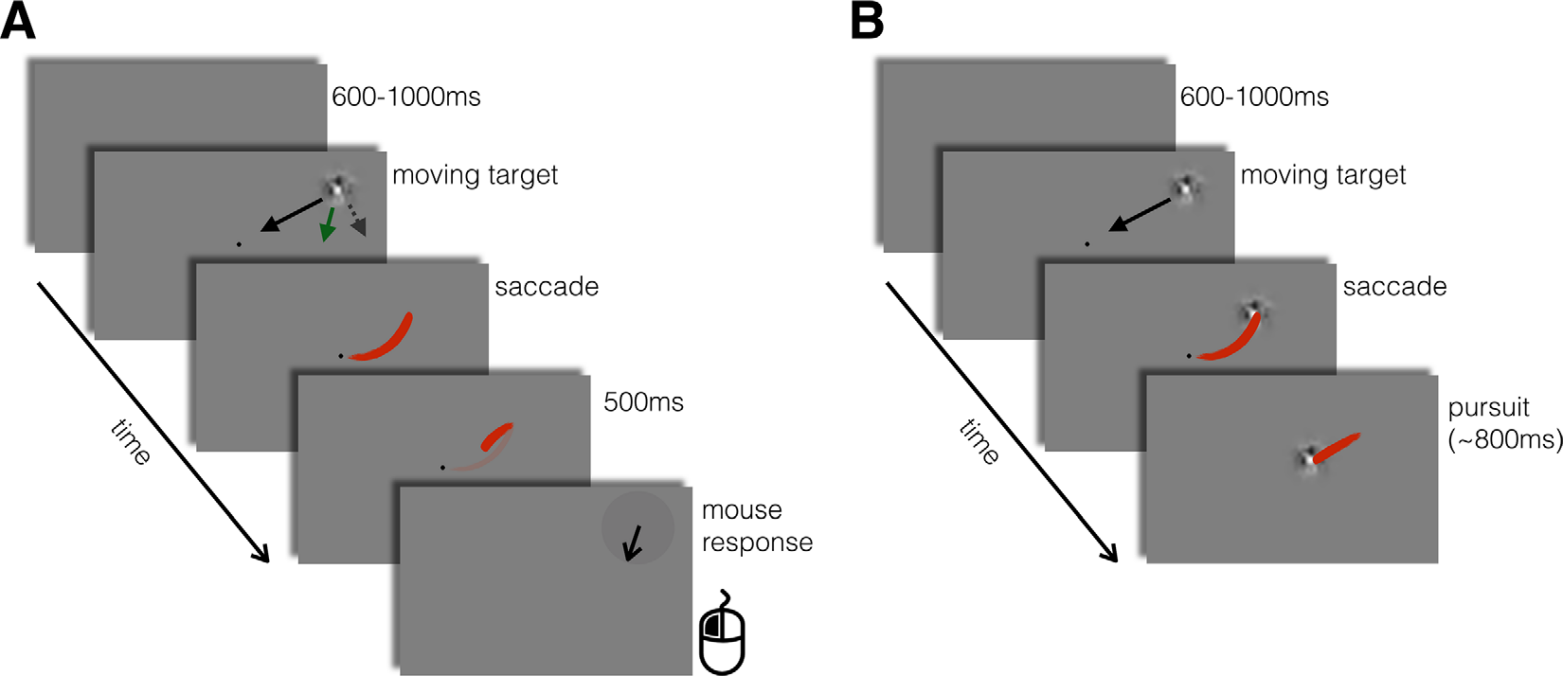
Experimental protocol. (**A**) Trial sequence in the double-drift condition. The red lines represent hypothetical gaze position traces, during and after the saccade. Even when the target was not displayed after the saccade landing, pursuit was initiated after the landing (Krauzlis & Lisberger, 1994). The black line indicates the physical motion of the envelope on the display, the dashed arrows the direction of the internal motion, and the green arrow the hypothetical perceived direction. At the end of the trial, the participant reported the direction of the moving target. (**B**) Trial sequence in the control condition. Here the target remained visible after the saccade and participants had to track it until it disappeared. These two conditions were randomly interleaved and difficult to distinguish prior to the saccadic response (verified by a subsequent control condition). Participants were never asked to report the perceived direction of the target in control condition; the purpose of the control condition was to provide a baseline for eye movement analyses and, by providing postsaccadic vision of the moving target, to make pursuit a default response upon saccade landing.

Each trial began when the participant’s gaze position was detected continuously within a circular area of 2 dva of diameter centered on the fixation point (a black disk of 0.2 dva of diameter) for at least 200 ms. After a random interval (uniformly distributed within 600–1,000 ms), during which the fixation point was continuously displayed, the stimulus appeared at a random position, moving either toward or away from fixation at 12 dva/s. The starting position of the target was defined on each trial by drawing a random angle (uniformly distributed in [0, 2π]) and adjusting the radius so that the target would (given its direction, inward vs. outward) reach an eccentricity of  8 dva at the time of saccade landing. The expected time required by the participant to complete the saccade was estimated on every trial by summing the average saccade latency of the previous 20 trials (assuming a value of 200 ms for the first 20 trials) with the expected duration of the saccade, which we estimated as 36 ms. The duration was estimated according to the formula 23.6 + 2.94*A*, (Abrams, Meyer, & Kornblum, 1989), where *A* is the saccadic amplitude and assuming a 10% amplitude undershoot (Becker, 1989; Lisi, Solomon, & Morgan, 2019). The moving target appeared and immediately started moving, and the participant was required to intercept it with a saccadic eye movement (see Figure 1).

Unbeknownst to the participants, on half of the trials, the target was a double-drift stimulus (double-drift condition), whereas in the remaining half, randomly interleaved, the target was a control stimulus (control condition). In addition to the different target stimulus (see Stimuli for details), the trial sequences of these two conditions also diverged after the saccade onset. On double-drift trials, the target was removed immediately after the saccade started (specifically as soon as gaze left a circular area of radius 2.5 dva around fixation) and was replaced after 500 ms by a black arrow (random orientation); in these trials, the participants were asked to report the direction of motion of the moving target by adjusting the direction of the arrow with the mouse (see Video 1 for some examples of double-drift trials). Importantly, even though there was no target present after saccadic offset in these trials, and therefore no visual motion signals, we expected to find a short (≈ 100 ms) postsaccadic pursuit response, corresponding to the open-loop response of the pursuit system (Krauzlis & Lisberger, 1994), which must have been driven by presaccadic velocity information.

**Video 1.** Video showing some examples of stimuli and recorded gaze position (red trace) in double-drift trials (Figure 1A). In the experiments, these trials were randomly interleaved with the control trials (shown in Figure 1B and in Video 2). Movie is available on the journal website.

On control trials, participants were not required to report the perceived direction. The target was a control stimulus (i.e., containing no internal motion; see Stimuli for details) that remained visible and continued moving after the saccade; participants were required to track the target with their gaze until it disappeared (see Video 2 for some examples of control trials). The purpose of the control condition was mainly to provide a baseline for eye movement analyses (in particular for saccade analyses). This design choice ensures that repeated postsaccadic disappearances of the target do not lead participants to form prior expectations about the lack of postsaccadic motion, which could attenuate the open-loop pursuit response (Krauzlis & Adler, 2001). Indeed, control and double-drift stimuli were designed to appear as similar as possible, so that participants would not realize that two qualitatively different types of stimuli were being presented. To test that this manipulation was successful, after the main task, we revealed that there were two types of stimuli and measured participants’ ability to discriminate the two types of stimuli (see Experiment 1: discriminability task for details).

**Video 2.** Video showing some examples of stimuli and recorded gaze position (red trace) in control trials (Figure 1B). Movie is available on the journal website.

The instructions given to the participants were to look at the moving object and track it with their gaze and, if the object disappeared, to report its direction of motion by adjusting the orientation of an arrow on the visual display. As mentioned in the Stimuli section, we designed control and double-drift stimuli so that the perceived temporal rate of change of their internal textures was as similar as possible. Furthermore, we introduced random deviation in the trajectories of control stimuli to make them similar to the trajectories of double-drift stimuli. Indeed, although the trajectories of double-drift targets were always physically aligned on a radial direction with respect to fixation, the perceived trajectories were shifted toward the direction of the internal motion (Kwon et al., 2015; Lisi & Cavanagh, 2015; Shapiro, Lu, Huang, Knight, & Ennis, 2010; Tse & Hsieh, 2006). In order to make the trajectories of control stimuli as similar as possible to the trajectories of double-drift stimuli, we introduced random shifts in their direction angle: Specifically, we rotated them around the expected interception point (at 8 dva eccentricity) by either adding or subtracting (with equal probability) a random angle uniformly distributed within 15–35° (this range was defined to include perceptual shifts observed in pilot tests with the same paradigm).

Trials in which participants blinked or moved their gaze from fixation before the appearance of the target were aborted and repeated within the same block. Each participant performed two sessions of the task, each comprising 400 trials split in four blocks. Participants were allowed to take a break whenever they felt the need. A standard 9-point calibration procedure for the eye movements was repeated after each break.

#### Experiment 1: Discriminability task

Given the short presaccadic stimulus presentation, it was difficult to distinguish between the two types of stimuli before the saccade. Indeed, after being debriefed, all the naive participants reported not having noticed any difference in the stimulus between the two trial types (i.e., between trials in which they should track the target after the saccade and trials where the stimulus disappeared after the saccade and they made a direction response). To make sure that the two conditions were not distinguishable, we ran each participant through a subsequent experiment. We explained to the participants that in the previous task, there were two different motion stimuli (control and double-drift) by showing static examples of each. Next we presented stimuli (generated with exactly the same procedure and parameters as in the main task) and asked them to maintain fixation and report, after the stimulus disappeared, whether the stimulus was a control or a double-drift stimulus (no saccade or pursuit required). The stimulus was presented for a variable duration, and after it disappeared, two static examples of the stimuli were presented on the left and right sides of fixation (see Video 3 for an example of the response display); participants provided the response by pressing either the left or the right arrow. The duration of the stimulus was adjusted online according to a weighted up-down staircase procedure that sought to find the duration of the target that yielded 75% correct responses. There were four independent staircase procedures, two for the inward direction and another two for the outward direction, starting at 100 ms and 800 ms of duration. The staircases were randomly interleaved, and each of them comprised 80 trials.

**Video 3.** Example of the response display used in the discriminability task of Experiment 1, showing an example of a double-drift target alongside an example of the control target. Movie is available on the journal website.

#### Experiment 2

Experiment 2 used the same procedure as the main task of Experiment 1, with the only difference that three, randomly interleaved, internal speeds or temporal frequencies were used. Each participant performed four sessions of the task, each comprising 480 trials split in eight blocks.

### Analysis

**Figure 2.**
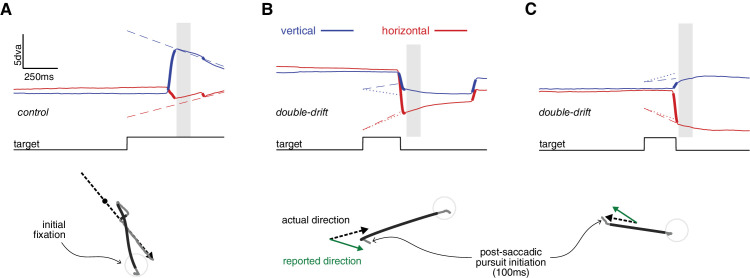
Example of eye movements traces (Experiment 1). (**A**) Example of a control trial: In the top panel, the horizontal (red) and vertical (blue) gaze (continuous line) and target (dashed lines) positions are plotted as a function of time. The thicker parts of the gaze position traces indicate saccades. In the bottom panel, the same trial is represented in x–y screen coordinates: The dashed black line indicates the trajectory of the target (the small dot indicates the position of the target at saccade landing time) and gray lines indicate gaze position (the darker parts indicate saccades). In this trial, the target moved inward from a peripheral position; the participant first intercepted it with a saccade and then tracked until it disappeared. The vertical gray square delimits the temporal window used for pursuit analysis (20–100 ms after saccade landing time). (**B, C**) Examples of double-drift trials. Notice that the target disappeared after saccade onset, but nevertheless pursuit was initiated after the saccade landing. The dotted lines in the top panels, and the green arrow in the bottom panels represents the direction that the participant reported at the end of the trial.

**Figure 3.**
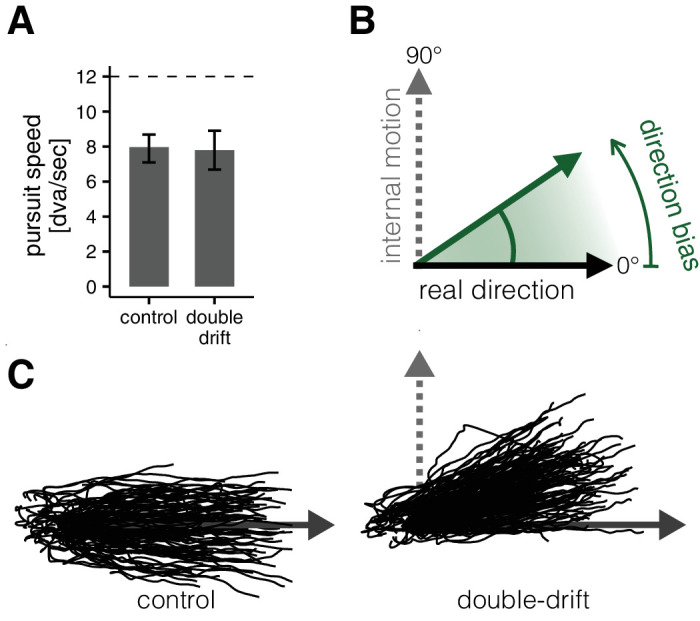
Experiment 1, data analysis: postsaccadic pursuit traces. (**A**) The average speed of pursuit in the postsaccadic interval (20–100 ms after saccade offset) was similar independently of the condition (control vs. double-drift) and thus independently of whether a moving target was actually displayed or not. Error bars indicate 95% CI. (**B**) Computation of the direction bias. Each postsaccadic pursuit trace was rotated so that the real direction of motion of the target was at 0° and the direction of the internal motion was at 90°; the same convention was used for the analysis of the direction bias in perception and saccade landings. (**C**) Raw postsaccadic gaze position traces from one participant (rotated according to the conventions illustrated in panel B); in the double-drift condition, the whole distribution of traces is pulled from the real direction of motion toward the direction of the internal drifting pattern.

**Figure 4.**
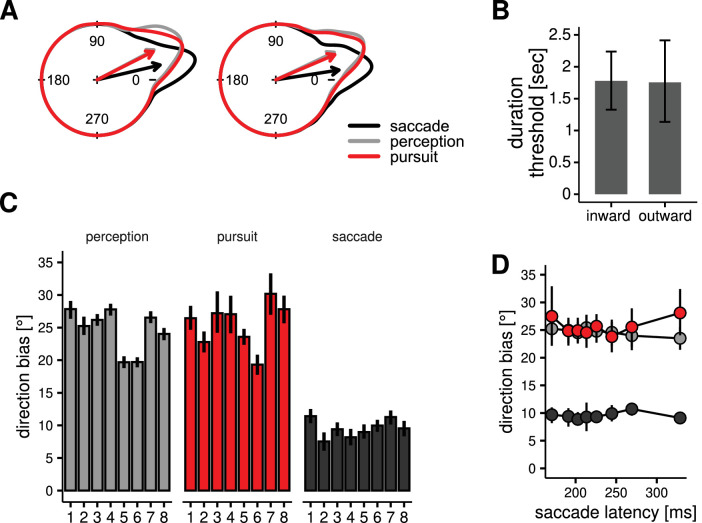
Experiment 1: results. (**A**) Examples of individual distributions of direction bias (same conventions as Figure 3B) for Participants 1 and 2. Arrows indicate mean direction biases. (**B**) Results of the discriminability task. The duration threshold is the minimum duration of presentation required to reliably discriminate (75% correct discriminations) the double-drift stimuli from the control stimuli in the fixation condition. Notice that the thresholds are much longer than the presentation duration in the main experiment (maximum 600 ms), indicating that participants would be at less than threshold discrimination levels in the main experiment. (**C**) Direction biases for all eight participants. Error bars represents bootstrapped 95% CI (percentile method). (**D**) The size of the directional bias was not modulated by the latency of the movement for saccade, pursuit, or perceptual responses (see text for details).

In the main task of Experiment 1 and in Experiment 2, we analyzed perceptual response and eye movement data to give, for each trial, three independent measures of the effect of the internal motion (in the double-drift condition): one from saccade landing positions, the second from the direction of the postsaccadic pursuit, and the third from the perceptual reports (see Figure 2). For each of these measures, we computed the angular differences between the reported/estimated direction, as well as the direction of the displacement of the target. To obtain a measure of direction bias induced by the internal motion, we transformed these differences so that positive value would indicate a deviation from the physical direction (external motion) in the direction of the internal motion (see Figure 3B). Examples of the distributions of angles obtained are reported in Figure 4A for two of the participants.

####  

##### Deriving the saccade system’s estimate of stimulus speed and direction.

Raw gaze position data were transformed to eye velocities and smoothed by using a moving average over five data samples (Engbert & Kliegl, 2003); saccade onsets and offsets were detected using an algorithm based on these 2D eye velocity (Engbert & Mergenthaler, 2006). Next, we estimated the direction of motion that was used by the saccadic system to intercept the target in double-drift condition. We computed a metric known as saccadic velocity compensation, SVC (Groh et al., 1997), by computing the difference between the spatial position of the saccade end point and the starting position of the target, and dividing it by the temporal interval from target onset until the end of the saccade. The SVC corresponds to the target velocity vector (speed and direction) for which the saccade would have been maximally accurate (i.e., for which the landing error would have been zero) and has also been referred to as the “position/time landing ratio” (Bourrelly, Quinet, & Goffart, 2018a). The variability of this measure arises mostly from the typical scatter of saccade landing positions (van Opstal & van Gisbergen, 1989) but might also reflect eye tracker inaccuracies, direction estimation biases (Krukowski & Stone, 2005), and other systematic motor biases. To correct systematic biases and improve the accuracy of the estimated SVC, we calibrated our analysis using control trials, where there was no internal pattern motion to distort the direction that would be taken into account by the saccadic system. For each participant and direction condition (outward vs. inward), we fit a number of multivariate linear models, with the sine and cosine of the target direction angle as dependent variables. In all, we tested 32 different models, all of them included as a predictor in a trigonometric polynomial for the SVC direction angle (of degree 1 or 2, depending on the model), plus one or more of the following variables: direction of the saccade (also represented through a trigonometric polynomial of degree 1 or 2), with or without interaction with the SVC, and interactions between the SVC components and the saccadic latency or amplitude. These models correspond to a circular–circular regression (Jammalamadaka & SenGupta, 2001) with multiple predictors. To avoid overfitting, we evaluated the out-of-sample predictive performance of each of the 32 models through a leave-one-out cross-validation procedure (see Supplementary Figure S1). The best model selected according to this procedure is the one that is expected to generalize better to an independent dataset (Geisser, 1975), so we used it to estimate the target direction taken into account by the saccade system in the double-drift condition. The final model included as a predictor the raw direction angle of the SVC vector in interaction with a trigonometric polynomial of degree 2 of the saccade direction angle. This latter component improved the fit of the model by including saccade biases, which are known to vary as a function of saccade direction (Rolfs, Knapen, & Cavanagh, 2010). The average cross-validated error of the best model (measured in control trials) was 11.36°, about half of the average direction error of the raw SVC measure on the same trials, 20.03°.

##### Estimating postsaccadic pursuit direction and speed.

Postsaccadic gaze position traces were low-pass filtered using a 100-point finite impulse response (FIR) filter with a cutoff at 40 Hz. We restricted the analysis to pursuit initiation, from 20 ms to 100 ms after the landing of the first interceptive saccade (we excluded the first 20 ms after saccade landing to prevent any contamination of our pursuit measures by potential inaccuracies in the identification of saccade offsets). This is within the time range of typical open-loop pursuit (Krauzlis & Lisberger, 1994), where postsaccadic visual signals have not yet had time to influence the ongoing pursuit. We fit these postsaccadic position traces with a line (orthogonal regression) to estimate the direction of the pursuit. Additionally, we differentiated these position traces to obtain a velocity trace, which we averaged over the whole 80-ms interval to get an estimate of the horizontal and vertical pursuit speeds. We took the Euclidean norm of this velocity vector as a measure of pursuit speed (irrespective of direction), which we used to compare the pursuit gain in the two conditions, control and double-drift (see Results). We used the same procedure to measure gaze velocity before the saccade, within an interval from 80 ms to 20 ms before saccade onset, to investigate whether we could detect presaccadic pursuit initiation (Lindner & Ilg, 2000) in our paradigm.

##### Perceptual estimate of stimulus direction.

We took the direction of the arrow, as set by the participants in double-drift trials, as the perceptual estimates of target direction. For each participant and condition, the size of the perceptual bias was computed by taking the angular mean of the signed angular differences between the arrow and the true direction of motion of the target.

##### Discriminability task.

We analyzed the proportion of correct identifications of the type of stimulus (double-drift vs. control) as a function of the duration of presentation, to determine the minimum duration required to achieve 75% of correct identifications. We fit the data separately for each direction (inward vs. outward), using a generalized linear mixed-effects model with a probit link function and a fully parametrized variance–covariance random-effect matrix, fit with R (R Core Team, 2021) and the lme4 (Bates, Mächler, Bolker, & Walker, 2012) library.

## Results

### Experiment 1

####  

##### Perceptual and pursuit responses reveal similar estimates of target direction.

The offline analysis of eye movements excluded 4% of the trials, due either to missing values in the gaze position trace or to the execution of a blink or an eye movement before the appearance of the target. Of the remaining trials, 0.53% were excluded because the saccade latency was either shorter than 100 ms or longer than 600 ms. Additionally, 0.78% of the remaining trials were excluded due to extreme saccade amplitude values, either less than 3 dva or larger than 20 dva; a further 0.55% were excluded due to saccade durations longer than 100 ms and 0.15% due to pursuit speed higher than 60 dva/s.

In the remaining trials, we found that, on average, the perceived direction reported by the participants was deviated by 24.57° (*SD* 3.23) from the physical direction toward the direction of the internal motion. This shift in the mean direction was significantly different from zero for each participant (see Figure 4), but it was about 25% smaller than what would be expected according to the simple sum of the two motion vectors (≈32°). There was no significant difference between the bias measured in trials with inward versus outward motion in the target, *t*(7) = 1.63, *p* = 0.15.

To examine how the pursuit system responded to the double-drift stimulus, we analyzed the early postsaccadic pursuit movement. The speed of the pursuit from 20 to 80 ms after saccade landing was on average 7.91 dva/s (*SD* 1.59), corresponding to a gain of 0.66 (*SD* 0.13) from the stimulus speed of 12.0 dva/s. The average speed in the control condition was 8.00 dva/s (*SD* 1.42) and 7.81 dva/s (*SD* 1.80) in the double-drift condition. A repeated-measures analysis of variance (ANOVA) did not reveal significant effects of condition (double-drift vs. control), *F*(1, 7) = 0.89, *p* = 0.38; direction of motion (inward vs. outward), *F*(1, 7) = 1.44, *p* = 0.27; or the interaction between condition and direction, *F*(1, 7) = 2.50, *p* = 0.16, on pursuit speed. This finding suggests that the eye movement behavior did not differ, in the first 100 ms after saccade landings, between the control and double-drift conditions, despite the fact that the moving target was present after the saccade landing only in the control condition (in the double-drift condition, the screen was blank after the saccade). We then restricted the analysis to the double-drift trials. We found that the direction of the pursuit was shifted on average by 25.25° (*SD* 3.30) from the physical direction of motion of the target, toward the direction of the internal motion. The size of the shift did not differ across inward versus outward trials, *t*(7) = 0.09, *p* = 0.93. The deviations of direction measured in pursuit were not different from those measured in the perceptual responses, *t*(7) = 0.76, *p* = 0.47 (see Figure 4C). Additionally, there was a tendency for a positive correlation across participants between pursuit and perceptual responses. The correlation was statistically significant with a one-tailed test, *r*(7) = 0.69, *p* = 0.03. We assessed also whether there were measurable correlations on a trial-by-trial basis. Correlations were quantified using a measure of circular dependence, which has the same properties of the product moment correlation coefficient for linear variables but is appropriate for angular variables (Jammalamadaka & SenGupta, 1988, 2001). In order to test statistical significance at the group level, individual correlation coefficients were transformed using Fisher’s Z transform (Fisher, 1915), which transforms the correlation coefficients such that their sampling distribution is approximately normal, and then their 95% confidence intervals were computed. For the correlation between perceptual reports and pursuit, the range was [−0.03, 0.19] with a mean of 0.10 and a 95% CI [0.04, 0.16]. For the correlation between saccade and pursuit, the range was [−0.14, 0.01] with a mean of −0.06 and a 95% CI [−0.10, −0.02]. For the correlation between saccade and perception, the range was [−0.08, 0.14] with a mean of 0.01 and a 95% CI [−0.05, 0.07]. Thus, although the correlation values were generally small (suggesting independent sources of noise affecting each response; see Supplementary Figure S5), we find some evidence for a correlation between perceptual reports and pursuit, suggesting a similar processing of motion information, and a negative correlation between saccades and pursuit, suggesting cooperative interactions between these two types of eye movements (Goettker et al., 2018; Goettker et al., 2019; Lisi & Cavanagh, 2017b).

##### Velocities underlying saccadic extrapolation differ from pursuit and perceptual velocity estimates.

Here we use the saccade landings to derive the estimate of stimulus velocity used by the oculomotor system to program the saccades. The average saccadic latency in the trials included in the analysis was 226 ms (*SD* 16) in control trials and 230 ms (*SD* 20) in double-drift trials; the latency did not differ across these two conditions, *t*(7) = 1.38, *p* = 0.21 (paired *t*-test).

In order to best estimate the target direction used by the saccadic system, we fit a model suited for angular data on the control condition, validated it through a cross-validation procedure (see Supplemental Figure 1), and then used it to estimate for each trial what was the most likely the target direction that elicited the observed saccades in the double-drift condition. The models were fit, on average, with 373 control trials per participant and were used to predict the direction of the target in (on average) 374 double-drift trials. With respect to control trials, the average *R*^2^ of the fit was 0.95 for horizontal (cosine) components and 0.92 for vertical (sine) components. This analysis revealed that in double-drift trials, the target direction estimated from saccade landings was on average shifted by 10.98° (*SD* 1.48) from the physical direction toward the direction of the internal motion, with no significant difference between trials with inward versus outward motion direction, *t*(7) = 0.85, *p* = 0.42. This level of direction bias was similar, although slightly smaller, than the one obtained by considering raw SVC measures, 13.55° (*SD* 2.96). Importantly, saccade responses were significantly less influenced by the internal motion than both perceptual, *t*(7) = 12.20, *p* < 0.0001, and pursuit responses, *t*(7) = 12.11, *p* < 0.0001 (Figure 4C).

We computed the absolute distance between the onset position of the target and the landing position of the saccade, divided by the temporal interval between target onset and saccade landing, and took it as a measure of the target speed (as opposed to direction). The average speed of the target estimated from saccade landing was very similar across control and double-drift conditions: 12.10 dva/s (*SD* 0.89) and 11.78 dva/s (*SD* 1.01), respectively, both very close to the actual target speed of 12.0 dva/s.

Finally, we investigated whether the observed difference between pursuit, perception, and saccades could be related to differences in the latency (see Figure 4D). We split the dataset into eight equally sized bins according to the individual saccade latency distributions. Thus, for each participant, each latency bin comprised 12.5% of their data ordered according to increasing saccade latency. For each response, we performed repeated-measures ANOVA with the observed direction bias as the dependent variable and the latency bin as the independent variable. This analysis failed to provide any evidence for modulation of the directional bias by latency in perceptual responses, *F*(7, 49) = 1.07, *p* = 0.40; pursuit responses, *F*(7, 49) = 1.23, *p* = 0.31; or saccade responses, *F*(7, 49) = 0.60, *p* = 0.75.

##### Presaccadic pursuit.

In Rashbass’s original studies (Rashbass, 1961), an initial pursuit response could be seen just prior to the saccade. To analyze any presaccadic pursuit here, we included in the analysis only trials where the speed of gaze movements in the presaccadic interval differed by more than 2 standard deviations from the gaze velocity measured in a baseline interval (300 ms before the onset of the target). The presaccadic gaze velocity exceeded this threshold only on 12% of the trials. We computed the difference between the direction of the presaccadic pursuit and the direction of displacement of the moving target, and performed a series of Rayleigh’s tests (Mardia, 1972) to assess whether these angular differences were concentrated along one direction or were uniformly distributed. The null hypothesis of circular uniformity was rejected (at the Bonferroni adjusted level α = 0.00625) in four cases out of eight in the control condition and in five cases in the double-drift condition. Overall, the average direction of the presaccadic pursuit roughly aligned with the external direction of the target envelope, in both the control and double-drift conditions (see Supplementary Figure S3), but due to the small number of trials and the large variability, it was not possible to determine whether the presaccadic pursuit was also significantly biased by the internal motion.

##### Perceptual similarity of control and double-drift stimuli in the discriminability task.

In the main experiment, the control trials, where the target remained after the saccade, were intermixed with the double-drift trials, where the target was absent after the saccade. The goal of this procedure was to encourage participants to make pursuit responses in the double-drift trials. In order for this strategy to be effective, the control and double-drift trials need to be indistinguishable. To examine this, we ran a separate experiment (discriminability task) in which we assessed the minimum duration of stimulus presentation required for the participants to reliably discriminate the two types of stimuli (control and double drift; see Stimuli section). Overall, distinguishing the two types of internal motion was quite difficult, and the duration yielding 75% of correct responses was much longer than the average saccadic latency (or duration of presentation) used in our main task (see Figure 4B). More specifically, the 75% threshold was estimated in 1,778 ms, bootstrapped 95% CI [1,326, 2,238], for inward moving targets and 1,755 ms, bootstrapped 95% CI [1,135, 2,414].

### Experiment 2

####  

##### Varying internal motion influences only estimated target direction, not speed.

In Experiment 2, we varied the speed of the internal drift, to gain some insight into how the internal and external motion signals are combined. We used the same rejection criteria as in Experiment 1, which led to the exclusion of 1.92% of trials due to saccade latencies either shorter than 100 ms or longer than 600 ms; 0.24% of trials due to saccade amplitudes shorter than 3 dva or larger than 20 dva; 2.99% due to saccade durations longer than 100 ms; and a further 0.08% due to pursuit speed higher than 60 dva/s. The average saccadic latency in the remaining trials was 222 ms (*SD* 43 ms) in control trials and 210 ms (*SD* 35 ms) in double-drift trials. We did not find evidence for a modulation of saccade latency due to the speed or temporal frequency of the noise, *F*(2, 6) = 2.17, *p* = 0.19; the condition (control vs. double-drift), *F*(1, 3) = 8,76, *p* = 0.06; or their interaction, *F*(2, 6) = 1.25, *p* = 0.35.

**Figure 5.**
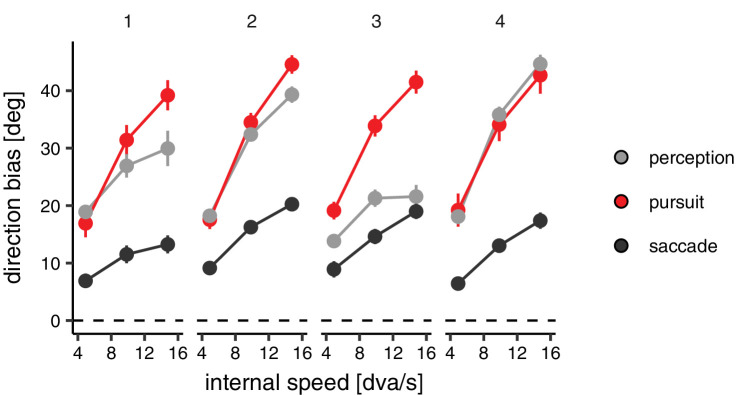
Experiment 2: results. Direction bias in perception, saccade targeting, and open-loop pursuit plotted as a function of the speed of the internal motion. Each panel represents data from one participant. Error bars are 95% confidence intervals.

The average directions reported by participants were shifted toward the direction of the internal motion by 17° (*SD* 2°), 29° (*SD* 6°), and 33° (*SD* 10°) for the conditions with internal speed set to 5, 10, and 15 dva/s, respectively (see Figure 5). The effect of the internal motion speed on the perceptual direction bias was statistically significant, *F*(2, 6) = 14.04, *p* = 0.005, whereas neither the effect of target direction (inward vs. outward), *F*(1, 3) = 7.253, *p* = 0.07, nor the interaction between direction and internal speed, *F*(2, 6) = 3.63, *p* = 0.09, reached statistical significance. The observed direction biases were again about 25% smaller than that predicted by a simple vector sum of the two motion vectors (≈ 22°, 36°, and 44°). Similarly, the analysis of pursuit responses revealed a strong effect of the speed of the internal motion, *F*(2, 6) = 128.4, *p* < 0.0001, but no evidence for an effect of target direction (inward vs. outward), *F*(1, 3) = 0.94, *p* = 0.40, or an interaction, *F*(2, 6) = 1.77, *p* = 0.25.

To analyze saccade responses, we used the same model selected for the analysis of saccade responses in Experiment 1. In this case, the model was fit on average on 899 saccades for each participant. The estimated direction biases revealed that saccade landings were also influenced by the speed of the internal motion, *F*(2, 6) = 85.56, *p* < 0.0001, but not by the direction of the target (inward vs. outward), *F*(1, 3) = 0.26, *p* = 0.64, or their interaction, *F*(2, 6) = 0.78, *p* = 0.50.

We then investigated whether the pursuit speed and the estimate of target speed recovered from saccade landings varied as a function of the speed of the internal motion. Indeed, if the integration mechanism was a vector averaging or vector sum, the increased speed of the internal drift should yield an overall faster speed estimate and therefore produce higher pursuit speeds (see Supplementary Figure S6) and saccade endpoints that are extrapolated even more along the direction of motion. However, the threefold variation of internal drift speed had no significant effect on pursuit speed or the speed estimate of the saccadic system (see Table 1): Pursuit, *F*(2, 6) = 1.61, *p* = 0.27, and saccades, *F*(2, 6) = 0.76, *p* = 0.51.

**Table 1.** Target speed (mean and standard deviations across observers) as inferred from the distance between target onset position and saccade endpoint and speed of postsaccadic pursuit, as a function of the speed of the internal motion.

Internal speed	Saccades	Pursuit
5 dva/s	11.92 (0.95)	6.65 (0.53)
10 dva/s	12.20 (1.19)	6.80 (0.61)
15 dva/s	12.06 (1.33)	6.66 (0.48)

In sum, the results of Experiment 2 revealed that the internal drift speed had a clear effect on the direction of the responses. However, it did not produce a significant effect on the speed estimates driving the eye movements.

## Discussion

In this study, we investigated the relationship between motion estimates used in saccades, pursuit, and perception. We presented moving apertures filled with noise that either drifted orthogonally to the aperture’s direction (a double-drift stimulus) or varied dynamically with no net motion (control stimulus). The first of these two, the double-drift stimulus, produces a remarkably robust deviation in perceptual judgments of direction of up to 45° or more (Lisi & Cavanagh, 2015; Shapiro et al., 2010; Tse & Hsieh, 2006). Using this stimulus, we previously demonstrated a dissociation between the perceptual and saccadic localization of moving objects (Lisi & Cavanagh, 2015). However, this initial report could not determine whether the dissociation originated from the differences in how motion signals are processed during the planning and execution of saccades and the formation of perceptual judgments, or from how these processes use these signals to extrapolate the position of the moving object. To address this question, in the current study, we designed a novel protocol to measure and compare the effects of the internal drift motion in perceptual judgments and two types of eye movements, saccades and pursuit, with all three measured on each trial in which a double-drift stimulus was presented. The targets appeared in the periphery and moved either toward or away from the central fixation point. Participants were asked to saccade to these targets as soon as they appeared and then track them with their gaze. Participants were instructed that in some trials, the target would disappear upon saccade landing, in which case they were required to report the direction of its motion using a directional arrow. In these trials, early postsaccadic pursuit (open loop) still occurred, and thus the presaccadic target velocity was reflected in the postsaccadic pursuit. This allowed for a trial-by-trial comparison of perceptual and pursuit responses. From the analysis of saccade landing position and time, we also estimated the direction and velocity of target motion taken into account by the saccadic system. Overall, the results showed that motion signals from the internal drift strongly affected not only the perceived target direction but also, with a similar magnitude, the direction of postsaccadic pursuit. This suggests shared motion signals in the formation of perceptual and pursuit oculomotor responses, in agreement with previous research showing that these two systems display comparable biases with a similar time course when solving the aperture problem (Beutter & Stone, 1998; Lorenceau, Shiffrar, Wells, & Castet, 1993; Pack & Born, 2001). In contrast, the effect on saccade landing positions appeared substantially smaller, less than half the shift observed in the perceptual and postsaccadic pursuit responses.

In Experiment 2, we varied the speed of the internal drift motion and observed changes in the direction of postsaccadic pursuit, but not in its speed. This indicates that the oculomotor system combines the internal and external motion components of the double-drift stimulus according to some integration process that is not simply explained by a weighted vector average (Heller, Patel, Faustin, Cavanagh, & Tse, 2021) (see Supplementary Figure S7). Saccadic responses showed a similar pattern. We calculated the target speed estimated by the saccade system by analyzing the saccade landing time and position relative to the target motion onset (see Analysis section). The saccade system compensates for target motion during the delay of programming and execution of the saccade by extrapolating the landing along the direction of target motion (Fleuriet & Goffart, 2012). We found that changing the internal speed of the target only influenced the direction of extrapolation, but not the amount of extrapolation. Previous studies have examined the effect of middle temporal visual area (MT) microstimulation on monkeys' oculomotor behavior during the tracking of a moving target (Groh et al., 1997) and found evidence for a vector averaging mechanism. In contrast, our results do not support simple vector averaging, suggesting that introducing a motion signal with MT microstimulation and introducing it with a drift within the moving target produce different effects. A plausible explanation for this observed pattern is that the brain maintains distinct velocity estimates for internal (pattern drift) and external (object displacement) motions, echoing the assumptions of a recent Bayesian object-tracking model (Kwon et al., 2015). This assumption is supported also by the finding that different biases, attraction or repulsion, can be observed depending on whether observers are asked to judge the direction of the object or of the internal pattern (Morgan, 2017). Yet, alternative explanations exist: For instance, the vector combination could assign different weights to direction compared to speed. Moreover, other information sources might influence speed estimation more than direction, such as position information, which is known to affect pursuit speed (e.g., Blohm, Missal, & Lefèvre, 2005; Goettker et al., 2019).

Overall, the pattern here of a larger influence of the internal motion for perceptual judgments than for saccadic eye movements aligns with our prior studies (Lisi & Cavanagh, 2015, 2017c; Massendari et al., 2018). However, there are some differences in the current data. Compared to earlier findings, saccadic errors here appear more akin to perceptual ones, revealing a reduction in the previously observed dissociation. A bias in saccadic targeting was also reported by Lisi and Cavanagh (2015) (see in particular their Supplementary Figure S2), but it differed from perception in that it was constant along the motion path and was smaller in size, consistent with the effects observed on static, drifting Gabors (Kosovicheva, Wolfe, & Whitney, 2014). One possibility that may explain why in the present study, the effects of internal motion on saccades are more similar to those on perception is the target’s motion path: While previously we examined vertical, tangential paths, in the current study, the trajectory is radial. A recent study of the double-drift in marmosets (Dotson et al., 2023) also used a radial trajectory and did find an effect of the illusion on saccade landings of approximately 20% of the perceptual illusion observed in human trials. While the radial versus tangential distinction remains speculative pending direct comparisons in identical subjects, our findings suggest that path orientation may modulate the weighting of internal and external motion components.

Another important distinction from previous studies is that here the perceptual and saccade responses occurred simultaneously on the same trials, raising interesting questions about the role of attention during the perceptual judgment. The established understanding is that attention is allocated at the future target location during saccade preparation (e.g., Kowler, Anderson, Dosher, & Blaser, 1995). Crucially, it is within this same interval that participants in our study form their judgment on perceived target direction. The observed discrepancies between saccadic and perceptual errors in our setup challenge the hypothesis that these differences arise due to the absence of presaccadic attentional shifts in perception-only trials (Nakayama & Holcombe, 2020). Instead, these results concur with evidence indicating that manipulating attentional resources scarcely affects the double-drift illusion (Haladjian, Lisi, & Cavanagh, 2018).

A potential interpretation of the observed differences between saccade and perception responses pertains to the role of spatial references. Spatial landmarks are an integral part of object localization (Deubel, 2004), attention allocation (Lisi, Cavanagh, & Zorzi, 2015), and saccade targeting (Li et al., 2017). The diminished bias in saccade targeting could potentially be attributed to an increased reliance on spatial cues from the screen edges. In our experiments, the visual display covered a substantial portion of the visual field (42 and 60 dva horizontally in Experiments 1 and 2, respectively). Meanwhile, targets were consistently around 8 dva from fixation during saccade landing, leaving a large gap (>13 dva) between the screen edge and postsaccadic gaze location. Landmarks at these distances have demonstrated modest yet consistent impacts on both memory-guided saccadic and perceptual localization (Camors, Jouffrais, Cottereau, & Durand, 2015; Li et al., 2017). However, these effects are anticipated to be even more subtle for continuously presented targets, as seen in this study. Consequently, it seems unlikely that differences in perceptual versus saccadic landmark effects could account for our findings. Nonetheless, conclusively ruling out this possibility requires repeating the experiments in an environment with no spatial references (other than the moving target), conditions that our current dataset cannot explore.

The different pattern of errors shown by perceptual judgments and saccadic eye movements here and in our previous studies (Lisi & Cavanagh, 2015, Lisi & Cavanagh, 2017c; Massendari et al., 2018) could be interpreted as due to a different processing of visual motion signals. For example, sensory maps representing local motion measurements (i.e., monkey areas MT or V5) may be read out for separate tasks according to different algorithms (Groh et al., 1997). Indeed, some lines of evidence suggest that visual motion analysis can be specifically tailored for each function it serves (Simoncini et al., 2012), so that when confronted with the same motion stimulus, different responses, such as perceptual reports and eye movements, can reveal large differences in the estimated parameters (speed or direction) of the motion (e.g., Glasser & Tadin, 2014; Spering & Gegenfurtner, 2007; Spering et al., 2011). However, we found here that another eye movement response, smooth pursuit, reveals estimates of motion direction that are virtually equivalent to those found for perceptual judgments (see also Maechler, Heller, Lisi, Cavanagh, & Tse, 2021), suggesting that both perception and pursuit employ similar computational mechanisms to estimate the velocity vector of the double-drift stimulus. A possible interpretation of our findings could suggest that saccades and pursuit are guided by separate representations of velocity- and position-related signals. However, this interpretation contradicts previous research, which has consistently shown a close correlation and cooperative interactions between the saccade and pursuit-related systems when tracking moving targets (Erkelens, 2006; Goettker et al., 2018; Goettker et al., 2019; Lisi & Cavanagh, 2017a). This body of research supports instead the alternative view that saccades and pursuit are likely driven by shared representations (Goettker & Gegenfurtner, 2021; Hainque, Apartis, & Daye, 2016; Orban de Xivry & Lefèvre, 2007; Schütz & Souto, 2011) and have similar functional organizations (Krauzlis, 2004) (although for counterarguments, see Bourrelly, Quinet, & Goffart, 2018b; Bourrelly, Quinet, Cavanagh, & Goffart, 2016; Orlando Dessaints & Goffart, 2023).

If saccadic eye movements and pursuit share common motion-processing mechanisms, how can we explain that the saccade landings were systematically closer to the physical target locations that what would be expected based on perceptual reports and the direction of pursuit? One possibility is that the saccadic system uses a velocity vector that is similar to that used by perception and pursuit but extrapolates target position for an interval shorter than the saccadic latency. Specifically, the direction bias measured from saccadic landing positions in our task could be consistent with an extrapolation of the target position along the direction of early pursuit for an interval of only about 110 ms (see Supplementary Figure S4). This temporal interval is smaller than the typical saccadic latency but aligns well with the so-called saccadic dead time observed in short saccades of 8–10 dva amplitude (e.g., Etchells et al., 2010; Lisi, Morgan, & Solomon, 2022; Ludwig, Mildinhall, & Gilchrist, 2007). This short interval right before a saccade’s onset represents a time during which processing delays prevent afferent information from influencing the upcoming movement (this may not hold for longer saccades, with amplitudes of 30 dva or more and exceeding 100 ms in duration, which can even exhibit online steering influenced by visual input; e.g., Gaveau et al., 2003). This account proposes that, due to their fast programming, saccadic eye movements may extrapolate target position over a shorter temporal interval than perception does. Consequently, when dealing with a double-drift stimulus, this results in smaller position errors compared to those incurred by perception. We propose that this could be the result of a dedicated oculomotor map that accumulates information and predicts ahead in time over a shorter interval than perception. In other words, the dissociation between saccadic responses and perceptual judgments would arise at the stage where motion signals are integrated with position signals to extrapolate future positions and could originate in the different lengths of the temporal intervals on which the extrapolation operates. This hypothesis is consistent with the proposal that neural processing across the brain is organized in a hierarchy of temporal scales (Heeger, 2017; Murray et al., 2014). This idea implies that various processes in the brain operate on distinct time frames, with more complex processes generally integrating information over longer durations. This concept could provide a mechanistic explanation to other action–perception dissociations, such as those where eye movements were influenced by perceptually suppressed stimuli (Rothkirch, Stein, Sekutowicz, & Sterzer, 2012) or are more sensitive than perception to brief perturbation in the speed of a moving target (Tavassoli & Ringach, 2010). Taken together, these findings support the idea that neural pathways responsible for rapidly redirecting our gaze operate on a faster time scale than those responsible for perceptual inferences (Lisi & Cavanagh, 2015; Lisi & Cavanagh, 2017c; Massendari et al., 2018).

## Supplementary Material

Supplement 1
