# Supplementary material for: Different extrapolation of moving object locations in perception, smooth pursuit, and saccades
Source: J Vis. 2024 Mar 28;24(3):9. doi: 10.1167/jov.24.3.9 (PMC10996402; doi:10.1167/jov.24.3.9)
Supplement: Supplement 1 [file jovi-24-3-9_s004.pdf]

Supplementary Figures for *Different extrapolation of moving object locations in perception, smooth pursuit and saccades*

Matteo Lisi
Department of Psychology, Royal Holloway, University of London, UK

Patrick Cavanagh
Department of Psychology, Glendon College, Toronto, CA
Department Psychological and Brain Sciences, Dartmouth College, Hanover, New Hampshire, US

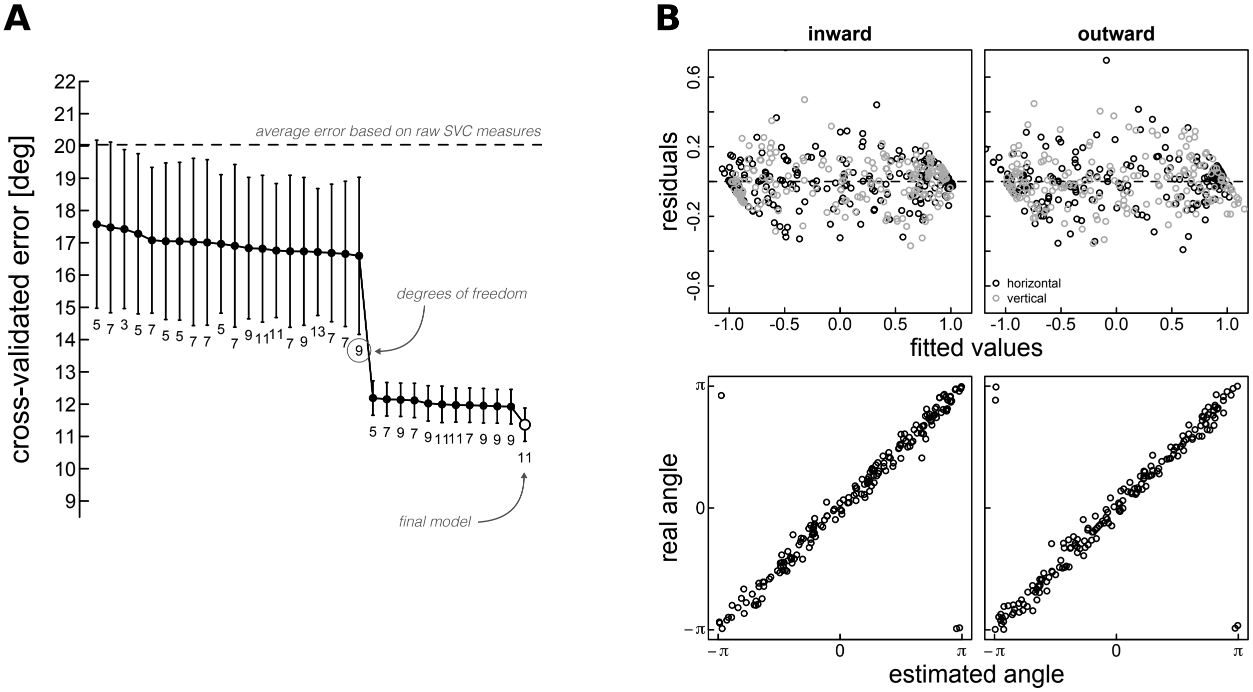

Figure S1: A. Each point represents a different multivariate model; the Y-axis represents the average error on the left-out trials (error bars represents the standard error across participants and conditions) measured in the cross-validation procedure. (Note that the model was fit to control trials where there was no bias on saccadic targeting and therefore we can assume that the direction taken into account by the saccadic system corresponded, except for internal noise, to the physical direction of motion.) This multivariate model was fit separately for each participant and direction (inward vs. outward). The horizontal dashed line represents the average error obtained by using the raw saccadic velocity compensation metrics (SVC) to estimate the target direction. The numbers underneath the dots indicates the number of degrees of freedom for each dimension (vertical and horizontal) of the models. The model used in the analysis presented in the main text is the one that obtained the smallest error and can be formally notated as

$$\begin{cases} \vec{V}_x = A_0 + A_1\vec{S}_x + A_2\vec{S}_y + \sum_{k=1}^{2}\left(A_{3k}\cos k\theta + A_{4k}\sin k\theta + A_{5k}\vec{S}_x\cos k\theta + A_{6k}\vec{S}_y\sin k\theta\right) + \epsilon_1 \\ \\ \vec{V}_y = B_0 + B_1\vec{S}_x + B_2\vec{S}_y + \sum_{k=1}^{2}\left(A_{3k}\cos k\theta + B_{4k}\sin k\theta + B_{5k}\vec{S}_x\cos k\theta + B_{6k}\vec{S}_y\sin k\theta\right) + \epsilon_2 \end{cases}$$

where $\vec{V}_x$ and $\vec{V}_y$ are the horizontal and vertical components of the target motion up to saccade landing; $\vec{S}_x$ and $\vec{S}_y$ are the horizontal and vertical components of the raw SVC metrics; θ the direction angle of the saccade, and ϵ_1, ϵ_2 normally distributed residual errors. We used this analysis to reduce the variance of the estimated trial-by-trial direction errors and increase the sensitivity of our analysis to trial-by-trial correlations between direction errors measured from saccades, perception and pursuit. We point out that analyses performed on raw SVC measures, or with different models all converged in showing the same pattern of results presented in the main text. B. Example of the model fit for one participant. The two panels show the residual error as a function of the predicted velocity components $\widehat{\vec{V}}_x$ (black dots) and $\widehat{\vec{V}}_y$ (gray dots); the lower panels show real and estimated values of target direction angle, calculated as $\arctan2\left(\widehat{\vec{V}}_y, \widehat{\vec{V}}_x\right)$ (where 'arctan2' denotes the four-quadrant inverse-tangent function).

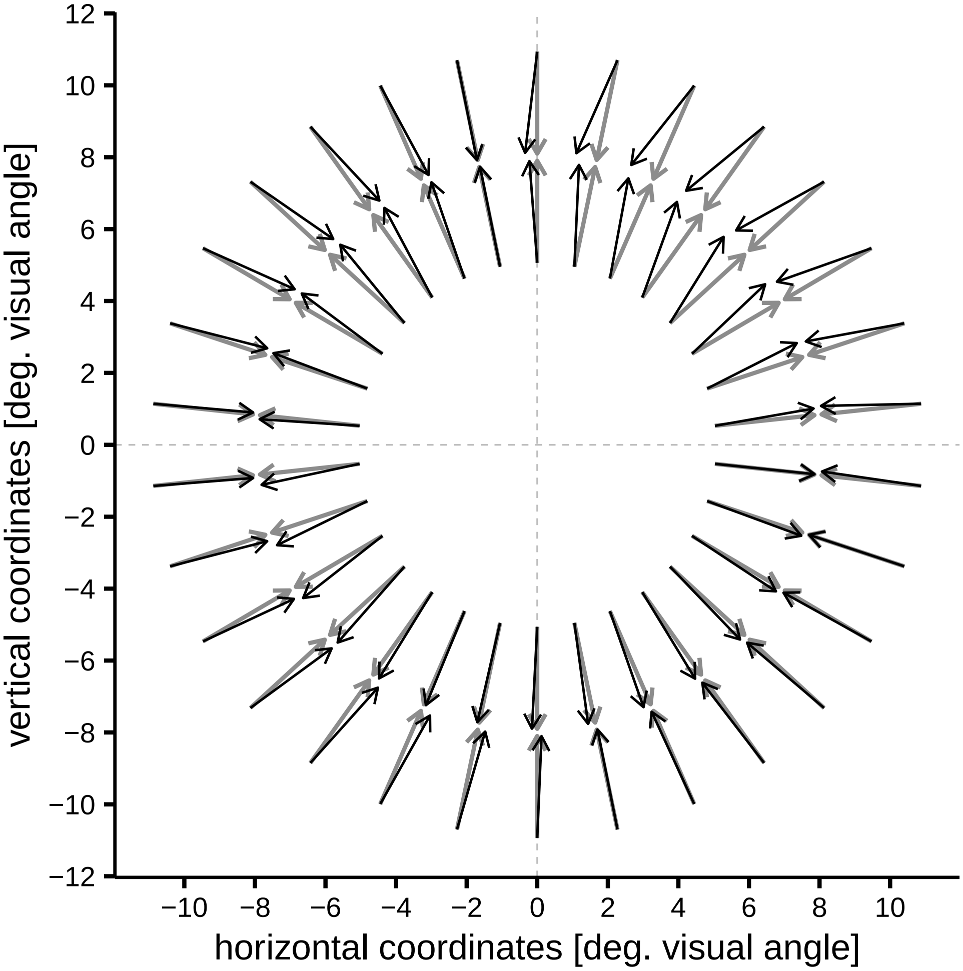

Figure S2: Example of correction of the estimated target direction from saccade landing. Raw saccade velocity compensation (SVC) measures can be affected by idiosyncratic, location-specific biases, possily due to changes in head position after the calibration or imperfect eyetracker calibration. This figure demonstrates how the model used in the analysis of saccade landings (see Main text and Fig. S1 for details) influence the estimated target direction, for one example participant. The grey arrows represents (hypothetical) raw SVC measures, shown for both inward and outward direction, and equally spaced around the visual field. The black arrows represents the predictions of the model, which was cross-validated on control trials to maximize the accuracy in predicting the target velocity vector. Note that the models for inward and outward saccades were fit independently (using data from different trials), yet they are highly consistent in how the vectors are displaced - see for example how the estimated vectors (black arrows) in the top-right quadrant are both shifted toward the left relatively to the raw measures (gray arrows). This suggests that the model-based estimates are correcting a calibration inaccuracy that affected the top-right part of the display, regardless of the target direction (inward vs. outward).

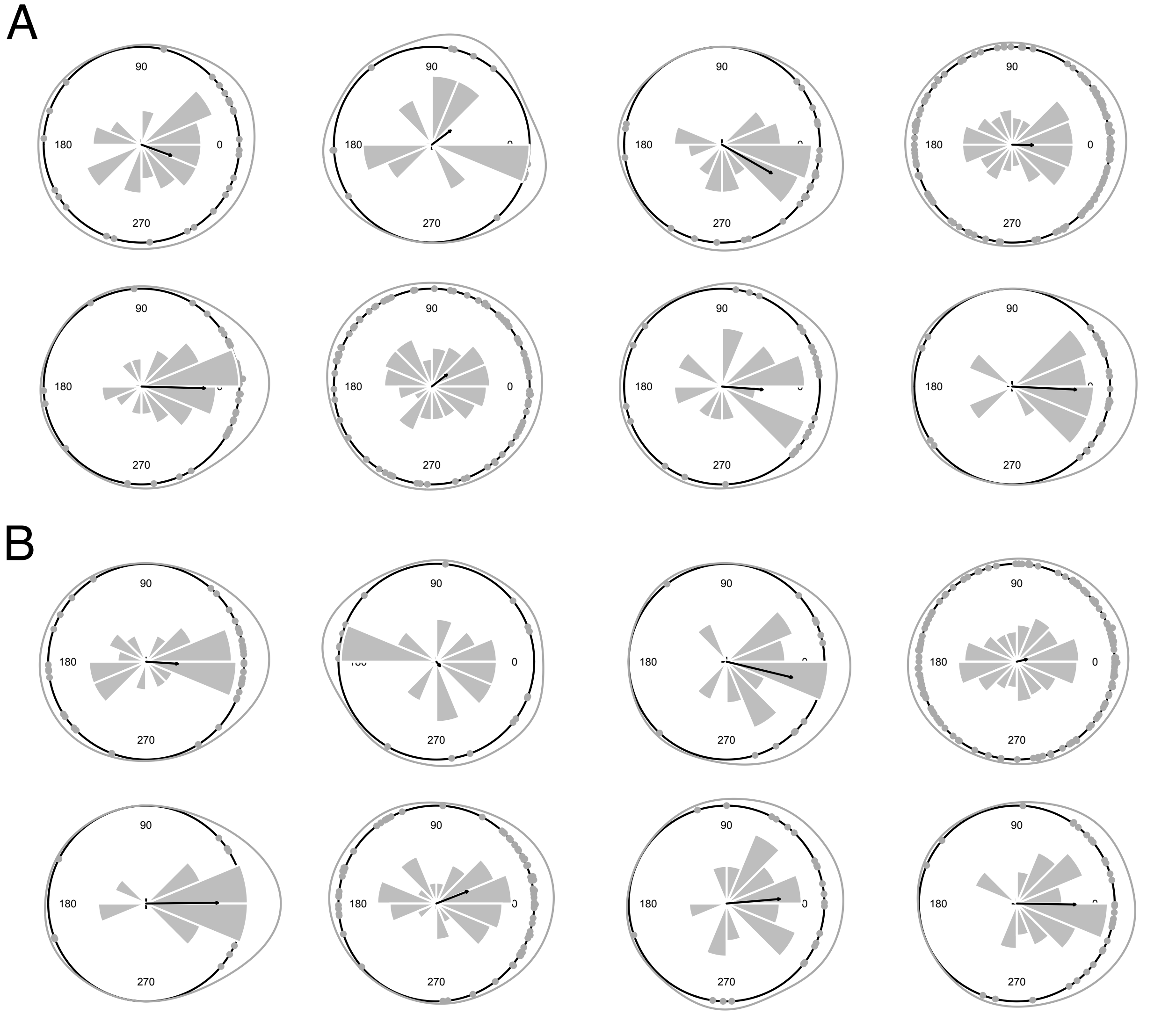

Figure S3: Direction of pre-saccadic pursuit. A. Control condition. Each point in the plots represents the direction of the pre-saccadic gaze movements on a given trial, with respect to the physical direction of the target envelope (which correspond in the plot to 0 degrees); the histogram and the circular density represent the distribution of these points, and the black arrow, the average direction. B. Double-drift condition. Using the same conventions as in the plots in the main text, a positive angular difference indicates a shift toward the direction of the internal motion. Only trials where the speed of the gaze movement exceeded 2 standard deviations of the gaze speed in the pre-stimulus baseline are included. These distributions suggest that there was, at least on some trials, a pre-saccadic pursuit response broadly tuned to the direction of the target. However, these pre-saccadic responses in our dataset were too small and variable to discern whether the internal motion of the target in the double-drift condition biased the responses.

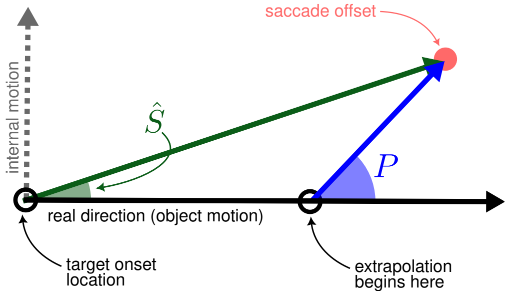

Figure S4: We estimated an extrapolation interval, corresponding to how far in time the saccadic system predicts future target locations. This analysis assumes that the saccade sistem extrapolates the target position using a biased motion equivalent to that driving early pursuit, and that this extrapolation begins only after a certain time has elapsed since the target onset. For each participant we found the interval t which minimizes the squared error between the observed direction bias, estimated by our analysis of saccadic landings, and the expected direction bias that would be obtained in the same analysis if the saccade extrapolated target position along the pursuit direction for an interval t^* (starting from an unbiased position). The expected saccadic direction bias $\hat{S}$ (shown as the green angle in the figure above) can be expressed as a function of the interval t and the pursuit direction bias P (shown as the blue angle in the figure above), $\hat{S} = \arcsin\left[\frac{\sin P \cdot \left(v \cdot t^*\right)}{v \cdot (\text{latency}+\text{duration})}\right]$, where v is the physical or external speed of the target, and latency and duration are the parameters of the saccadic eye movements. We found values of t^* numerically using data of the Experiment 2, separately for each participant; the mean value of t^* was 110 ms and the standard deviation across observers was 2 ms.

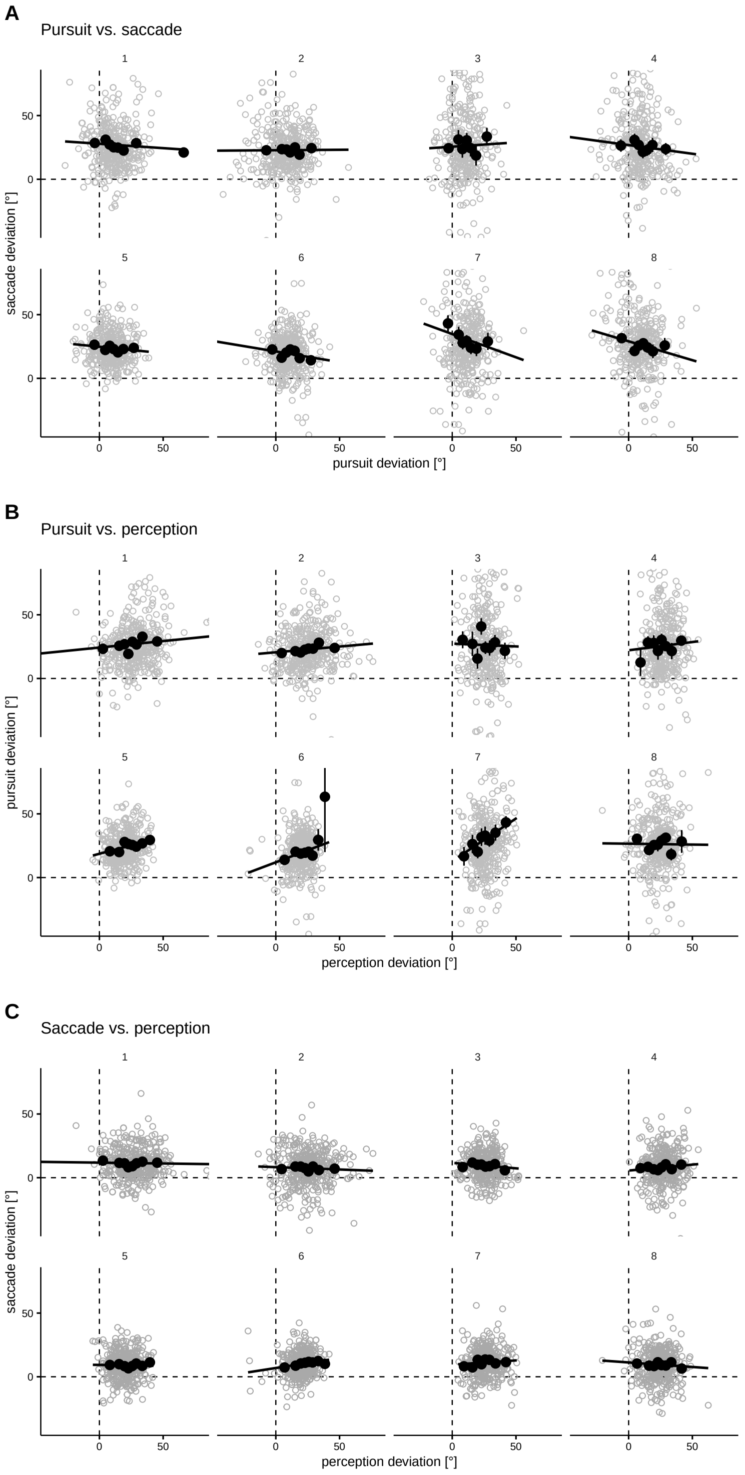

Figure S5: Relationship between trial-by-trial measures. Each panel illustrates a correlation betwen two of the variables, plotted separately for each observer in Experiment 1. Gray dots are individual trials; black dots are averages binned according to quantiles of the variables plotted in abscissa; and black lines are ordinary linear regression fits.

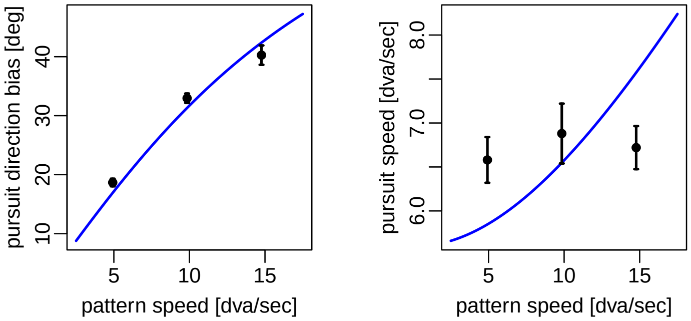

Figure S6: Discrepancy between the observed pursuit velocity in Experiment 2 and the predictions of a weighted average model (Heller et al, 2021). Blue lines represent model predictions, while black dots accompanied by error bars depict the observed data (averaged across observers) and their respective standard errors. The relative weights, denoting the ratio of internal to external motion weighting, were estimated to match the bias in pursuit direction (left panel). Additionally, we introduced a parameter that determines the gain of smooth pursuit. This was estimated by minimizing the mean squared difference between the predicted and observed speeds (right panel). The right panel emphasizes the mismatch between the predicted steep increase in pursuit speed with rising internal speed, predicted by the weighted average model fit on direction data, and the actual observed pursuit speed.
